# Angiogenic Cell Precursors and Neural Cell Precursors in Service to the Brain–Computer Interface

**DOI:** 10.3390/cells14151163

**Published:** 2025-07-29

**Authors:** Fraser C. Henderson, Kelly Tuchman

**Affiliations:** 1 Department of Neurosurgery, University of Maryland School of Medicine, Baltimore, MD 21201, USA; 2 The Metropolitan Neurosurgery Group, 1401 Mercantile Lane Suite #341, Upper Marlboro, MD 20774, USA; kelly@metropolitanneurosurgery.org

**Keywords:** ACP, NCP, progenitor cells, stem cells, NF-κB, NK cells, brain/AI interface, M2 phenotype, IL-8, learning, P75NTR

## Abstract

The application of artificial intelligence through the brain–computer interface (BCI) is proving to be one of the great advances in neuroscience today. The development of surface electrodes over the cortex and very fine electrodes that can be stereotactically implanted in the brain have moved the science forward to the extent that paralyzed people can play chess and blind people can read letters. However, the introduction of foreign bodies into deeper parts of the central nervous system results in foreign body reaction, scarring, apoptosis, and decreased signaling. Implanted electrodes activate microglia, causing the release of inflammatory factors, the recruitment of systemic inflammatory cells to the site of injury, and ultimately glial scarring and the encapsulation of the electrode. Recordings historically fail between 6 months and 1 year; the longest BCI in use has been 7 years. This article proposes a biomolecular strategy provided by angiogenic cell precursors (ACPs) and nerve cell precursors (NCPs), administered intrathecally. This combination of cells is anticipated to sustain and promote learning across the BCI. Together, through the downstream activation of neurotrophic factors, they may exert a salutary immunomodulatory suppression of inflammation, anti-apoptosis, homeostasis, angiogenesis, differentiation, synaptogenesis, neuritogenesis, and learning-associated plasticity.

## 1. Introduction

The research and development of brain–computer interfaces (BCIs) has flourished in the past several years, offering hope to individuals suffering from a myriad of conditions including paralysis, ALS, blindness, and Parkinson’s. BCI implants from several companies have allowed paralyzed patients to control robotic arms, write emails, and play chess with their thoughts. A non-verbal man with ALS recovered his voice with a Neuralink implant, and a Prima implant gave blind individuals “artificial vision”, allowing them to perceive letters and shapes. The BCI converts electrical signals from the brain into computer code which can be used for applications such as moving and clicking a mouse cursor. Non-invasive modalities are disadvantaged by weaker brain signals which require expensive amplification hardware and sophisticated signal processing techniques [1].

Invasive BCIs have better signal quality. However, electrodes implanted into the brain cause inflammation, scarring, and the loss of neurons. There is an increased risk of infection and rejection of the implant as a foreign entity [1,2]. Perpetual inflammation following implantation lasts for several months following insertion, can damage surrounding tissues, affect function, and may cause failure of the implant [3]. Recordings historically fail between 6 months and 1 year; the longest BCI in use has been 7 years [4]. A more pervasive problem is that implanted electrodes cause glial scarring and the encapsulation of the electrode. This is the result of activation of the microglia, which constitute 10% of brain cells, and the subsequent release of inflammatory factors and the recruitment of systemic inflammatory cells to the site of injury [5]. The scarring is exacerbated by the migration of meningeal fibroblasts along the electrodes [6]. These cells and activated glia eventually encapsulate the electrode with scar tissue and interfere or prevent signal transmission [7].

While the alacrity and skill of electrode placement and the quality of the electrode are significant positive variables affecting the quality of the electrode–brain interface, the immune response and triggering of programmed cell death—apoptosis—are presently the limiting factors to the long-term success of BCIs. The robotic placement of the implants and the micro-scaling of the electrodes will minimize these risks. However, as deeper electrodes are contrived, the surface area over the electrodes will necessarily increase, and ultimately result in some degree of microglial and astrocytic reaction, inflammation, scarring, apoptosis, and decreased signal transmission. Moreover, neurogenesis is limited in adults [8].

Of immense importance then is the development of a cell substrate that provides a direct contact interface between brain and computer. The development of brain organoid-based systems has inspired the combination of brain cells integrated with a biocomputer to accomplish simple tasks. There has been a flurry of interest in the use of cells to affect the microenvironment.

A biohybrid strategy incorporating cells as an intermediate layer on electronics allows for a “controllable” synaptic integration between implanted cells and existing neural circuitry. Biohybrid implants have the potential ability to host, interact, and control the behavior of transplanted cells, promote organized, functional cellular integration with living tissue, and reduce scar tissue formation (i.e., foreign body reaction) [9].

Biohybrid neural interface devices are bioelectronic technologies that utilize tissue engineering to achieve the goals of enhancing signal transduction and minimizing immune responses.

Biological tissue is a more natural connection between the device and the target tissue; biohybrid technologies provide living cells to affect the microenvironment of implanted electrodes, thereby influencing surrounding neural cell density. Various arrays fabricated with a hollow well to contain neural stem cells (NSCs) and containing alginate hydrogel may support host neuronal survival and reduce the tissue response through secreted neuroprotective factors [10]. The addition of hydrogel substrates to incorporate neural cells offers mechanical compliance, an ECM-like microenvironment that supports graft cell survival and the long-term functionality of the electrode surface. Hydrogels can thus create a microenvironment conducive to cellular growth and viability [11].

However, biohybrid neural interface device technology is yet nascent. Significant hurdles exist: the survival time of cultured cells, the optimal delivery of the cells to the transplantation site in the brain, and the software challenges of decoding brain signals.

Autologous induced pluripotent stem cells have been used in this context for the treatment of various neurological disorders, as they induce no foreign body reaction. However, they have low viability after transplantation and carry a risk of tumor formation [12]. NSCs secrete neurotrophic factors that promote axonal regeneration in the host neurons while at the same time reducing glial formation and enhancing healing [10]. NSCs have been cultured along a probe and showed growth and differentiation. The probe implanted into a rodent brain demonstrated a decreased glial reaction. The decreased gliosis was attributed to released neural growth factors (NGFs) [13].

More recently, living columns of cells have been introduced into the brain as an axon-based electrode consisting of a neuronal tissue engineered construct, acting as a transducer between the host tissue and an external electrical or optical neuromodulation device [14]. The “living electrode may have excitatory, inhibitory, or modulatory effect (glutamatergic, GABAergic, or dopaminergic) depending upon the type of synapses formed with the host. The goal is a biologic interface with high specificity, high synaptic density, and long-term integration. The length of the axons can vary between submillimeter to one centimeter [15]. Implanted living electrodes have been found to be viable at 1 month, with the appearance of synapse formation with host neurons [16].

As stem cell science and the BCI converge, these complimentary technologies offer solutions to profoundly impaired lives. However elegant the development of ‘living electrodes’, the problem of nature’s response to the foreign body—inflammatory signaling, altered excitotoxicity, loss of neural plasticity, neuron apoptosis, and loss of neurotrophic factors—will need to be addressed, and on a recurring basis. This requirement is met by a cellular milieu expressing neurotrophic factors that address inflammation and promote robust cell survival. Given the goal of neural signaling—and ultimately machine learning—the cell interface should be characterized by molecular activity that is known to directly promote neural plasticity.

While there are no published studies that examine the efficacy of angiogenic cell precursors (ACPs) and neural cell precursors (NCPs) in the context of the BCI, the extant literature supports a role for ACPs and NCPs and their distinctive attributes in the promotion of cell survival, reduction in scarring, and importance in neural plasticity. Particular attention is drawn to the high levels of IL-8 expressed by ACP, and the resulting draw of NK cells, and the downstream activation of NF-κB in its role as a transcription protein of central importance in neuronal plasticity and ultimately the provision of the anatomic substrate of machine learning.

## 2. ACPs Attract NK Cells and Exert a Modulatory Effect on Inflammation and Scarring

ACPs are derived from the synergetic cell, defined by the expression of CD31Bright, CD34+, CD45 Dim, and CD34Bright, but not lineage-specific features. The synergetic cell can differentiate into ACPs, NCPs, or myocardial cells depending upon exposure to defined culture conditions. ACPs express CD34+, CD133, KDR, Tie-2, CD144, von Willebrand factor, CD31Bright, the concomitant binding of Ulex-Lectin, and the uptake of acetylated low-density lipoprotein. ACPs express high levels of IL-8 (CXCL8), vascular endothelial growth factor (VEGF), and angiogenin, and form tube-like structures in vitro.

ACPs have a profound modulatory effect. In preclinical studies, ACPs have been shown to home toward the cytokine CXCL12 released by injured tissue, to embed [17], and to release angiogenic and growth factors.

**Figure 1:**
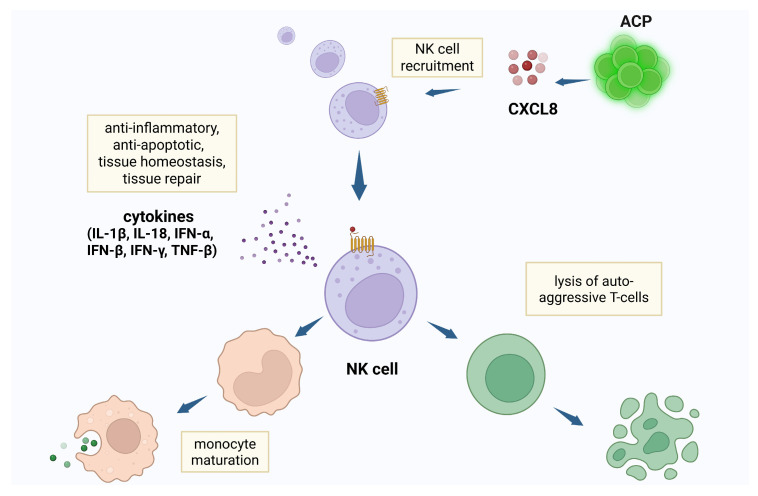
Natural killer (NK) cells recruited by angiogenic cell precursors (ACPs) suppress inflammation through release of anti-inflammatory cytokines, dendritic cell and monocyte maturation, and lysis of auto-aggressive T cells. Created in BioRender. Tuchman, K. (2025) https://BioRender.com/6rfoaiv.

The high level of ACP secretion of CXCL8 exhibits specific chemokine activity for NK cells [18,19,20]. CXCL8 is the most potent human neutrophil-attracting chemokine, playing a dominant role in response to infection and tissue injury, the activity of which depends upon interaction with human CXC chemokine receptors CXCR1 and CXCR2 [21]. CXCR1/2 receptors—expressed on NK cells, myeloid-derived epithelial cells, and other cell types [22]—are highly specific to CXCL8 [23,24]. Moreover, CXCL8 exhibits a systemic effect that extends to remote populations of NK cells (Figure 1). As part of the innate lymphoid cell population, NK cells suppress inflammation through receptor—ligand interactions and cytokine secretion. NK cytokine release alters Th subtype, lysing auto-aggressive T cells, and accelerating the maturation of monocytes and dendritic cells [25,26]. NK cells express IFNγ—a potent immune-modulator—and other mediators which create an anti-inflammatory chemokine environment that inhibits the assembly of inflammatory cells, limiting both fibrosis and apoptosis [27,28]. NK cells serve in the first line of defense against intracellular pathogens through the expression of type I interferons—IFN-α, IFN-β, and IFN-γ—enhancing the expression of major histocompatibility complex molecules of antigen-presenting cells, and initiating an anti-viral inflammatory cascade, preventing viral replication by detecting and destroying the infected resident cells [29,30]. NK cells are protective against pathogens—such as coxsackievirus B and cytomegalovirus—causing meningitis, encephalitis, seizures, and myelitis.

## 3. NF-κB Activated by IL-8 (CXCL8) in the CNS More Commonly Inhibits Apoptosis and Regulates Cell Survival

Though NF-κB is more commonly associated with inflammation and apoptosis, the authors emphasize the more complex and important role played by NF-κB in the central nervous system (CNS). Activated by the downstream effects of CXCL8, NF-κB may be pro-inflammatory depending on cell type and pathological state. Under the influence of NF-κB, microglia and glial cells in trauma produce reactive oxygen species (ROS) and pro-inflammatory cytotoxins [31], and can induce apoptosis in response excitotoxins, nitric oxide species, and certain death-inducing signals, depending upon the type and duration of stimulus. Alternatively, NF-κB proteins can promote the expression of anti-apoptotic genes.

In nerve cell populations, the NF-κB influence is usually protective [32]. In neurons, NF-κB induce genetic coding for anti-apoptotic proteins, neurotrophic factors, antioxidant enzymes, and calcium-regulating proteins. In the CNS, NF-κB thus generally functions to inhibit the apoptotic cascade; conversely, blocking NF-κB results in apoptosis. For instance, the inhibition of NF-κB in macrophages results in the release of Cytochrome C, an activator of the pro-apoptotic caspases [33,34,35,36,37]. These anti-apoptotic effects are especially demonstrated in sympathetic and sensory neurons, encompassing the sequestration of reactive oxygen species and enhanced neurite growth [38,39,40,41,42,43,44,45,46,47,48,49,50]. The activation of the NF-κB–MnSOD pathway confers a reduction in oxidative stress by the mitochondrial sequestration of free radicals [51]. Neuronal target genes encoding for neuronal survival include the neuronal apoptosis inhibitory protein, calcium calmodulin kinase II δ, (CaMKII δ), brain-derived neurotrophic factor (BDNF), μ-opioid receptors, neural cell adhesion molecule, manganese superoxide dismutase (MnSOD), Bcl-2, and calbindin D28k [52]. Cultures of rat hippocampal cells and sympathetic neurons subjected to excitotoxic or metabolic stress demonstrated increased survival, attributed to the neuroprotective effects of upregulated NF-κB and the induction of MnSOD [53]. NF-κB also induces the expression of cellular inhibitors of apoptosis, Bcl-2s, TRAF1/TRAF2, and may decrease the expression of apoptosis-promoting cytokines such as TNFα and FAS ligand [54,55,56,57,58].

The NGF induction of NF-κB is essential to the normal development, function, and maintenance of cells in the nervous system. The activation of NF-κB by NGF is well characterized, in part mediating effects in the nervous system through the activation of the p75 neurotrophin receptor (p75NTR). Generally apoptotic, P75 neurotrophic factor expression in the presence of a neurotrophin leads to programmed cell death through the caspase and JNK pathways. However, in the presence of tropomyosin kinase A, the transcription of which is promoted by NF-κB, P75NTR facilitates neurotrophic function by increasing the affinity of NGF binding to the tropomyosin kinase A receptor [59,60], and thus the aggregate of NF-κB signals exert neuroprotective effects [45].

## 4. Neural Cell Precursors (NCPs)

NCPs are generated from autologous peripheral blood by culturing the cells in medium supplemented with autologous serum, followed by activation in a defined medium containing specific differentiation-inducing factors. The cells acquire a neural phenotype characterized by the development of neuroglial morphology with neurite-like extensions and the expression of neural markers (Nestin, Beta-tubulin III, O4, and glial fibrillary acidic protein), and secrete neurotrophic factors including BDNF, NGF, glial cell-derived neurotrophic factor (GDNF), macrophage colony stimulating factor, and stem cell factor. In addition to the characteristic markers of neural lineage, the NCPs respond to neurotransmitters glutamate and GABA, as detected by the flow of Ca^2+^ through the voltage-gated Ca^2+^ channels. Preclinical studies have demonstrated the migration of NCPs to the penumbra of ischemic lesions, and to sites of laser-induced retinal injury [61,62,63,64].

While there are no published preclinical or clinical studies of NCPs in the context of the BCI, there is emerging evidence in preclinical studies and clinical trials that confirms the efficacy of stem cell therapy in stroke patients and other neurological disorders. However, the mechanisms through which stem cell grafts promote neural repair remain incompletely understood [65,66,67,68].

The therapeutic benefits of transplanted NCPs reside predominantly from the paracrine release of bioactive molecules, as opposed to the integration of the cell into injured tissue. This “bystander” effect may be the most important in stem cell therapy [69]. Robust expression of neurotrophic factors: BDNF, GDNF, Stem Cell Factor and Macrophage Colony Stimulating Factor is neuroprotective, promoting myelin formation, enhancing axon survival through interaction with the tyrosine kinases, p75 Neurotrophic factor, and NF-κB [70,71]. These paracrine mechanisms, including the release of other bioactive molecules, cytokines, and extracellular vesicles, modulate inflammation, promote neuroprotection, enhance angiogenesis, and stimulate endogenous repair processes in the injured brain.

## 5. Neural Cell Precursors May Mitigate the Pathophysiological Effects of CNS Injury Through the Modulation of the Expression of the N-Methyl D-Aspartate (NMDA) Receptors

The neurotrophins expressed by NCPs exhibit a salutary downregulation of NMDA receptors, which may be of importance in subjects with traumatic brain injury or post-traumatic stress disorder. N-Methyl-d-Aspartate (NMDA)-type receptors are ligand-gated ion channels which mediate excitatory transmission. Comprised of a diverse array of tetrameric receptor complexes that exhibit regional specificity, NMDA receptors exhibit distinct physiological roles across different neuronal cell types and brain regions. The sequence variants of NMDA receptors are associated with autistic spectrum disorder, developmental delay and intellectual disability, epilepsy, alterations of muscle, language problems, and other phenotypic manifestations [72]. NMDA receptors are pathologically upregulated in central nervous system injury, variably resulting in neural supersensitization, behavioral change, epilepsy, or apoptosis. Glutamate binding to NMDA in the setting of CNS injury results in Ca^2+^ influx and direct excitotoxicity, the release of apoptosis-inducing factor, and cell death. The downregulation of NMDA receptors by NCPs mitigates calcium-dependent neuronal injury [73], decreasing excitotoxicity and central sensitization [74].

## 6. The CXCR4/CXCL12 Axis Promotes ACP and NCP Migration Toward Areas of Ischemia and Injury

ACPs exhibit the robust expression of cell surface receptors CXCR1, CCR2, and CXCR4, facilitating the passage of cells across the blood–brain barrier [75,76,77]. Chemokine CXCL12, also known as Stromal-derived factor 1, is upregulated in neurons and glial cells following injury and is released in areas of CNS injury. CXCL12 induces the migration and activation of hematopoietic progenitor cells and endothelial precursor cells [21]. ACPs highly express the CXCR4 receptor, and migrate toward higher concentrations of CXCL12, repopulating injured CNS tissue [78]. NCPs also highly express the CXCR4 receptor [79] and migrate toward higher concentrations of CXCL12, repopulating injured CNS tissue [78]. Chemokine CXCL12 gene therapy attracts NCPs, endothelial progenitor cells, and oligodendrocyte progenitor cells to the injured sites of the brain promoting angiogenesis, neurogenesis, remyelination, and fostering the adoption of the non-inflammatory (M2) phenotype of monocytes [80,81,82].

NCPs are specifically programmed to differentiate into neural cells, astrocytes, and oligodendrocytes, and have demonstrated meaningful repair of injured brains. Exogenous stem cell transplantation has been shown to accelerate immature neuronal development in damaged brain regions [83], and to engraft, differentiate into oligodendrocytes, and contribute to the re-myelination of damaged axonal functions, facilitating recovery after spinal cord injury [84,85].

The extent to which the homing influence of CXCL12 exerts upon ACPs/NCPs in the context of the BCI is unknown. The degree to which chemotactic factor CXCL12 is expressed at the BCI reasonably relates to the degree of cell injury and ischemia resulting from the effects of electrode implantation, and the activity of the signaling, both in the acute and the chronic circumstances. Ultrasound, magnetic and electric fields, and irradiation have been used to increase homing to damaged tissue and could be used in conjunction with intrathecal injection to maximize cells targeting the BCI [86].

While the homing and replacement of neural cells, and the eventual circuit reconstruction may yield long-term improvements, the important initial gains in function are due to paracrine support enhancing neural plasticity [87].

## 7. Angiogenic Cell Precursors (ACPs) Promote Angiogenesis

CD34+ stromal cells are essential to angiogenesis, participating in cell migration, the control and organization of the extracellular matrix, scaffolding, immunomodulation, neurotransmission, the control and regulation of other cell types, and regeneration [88,89,90].

**Figure 2:**
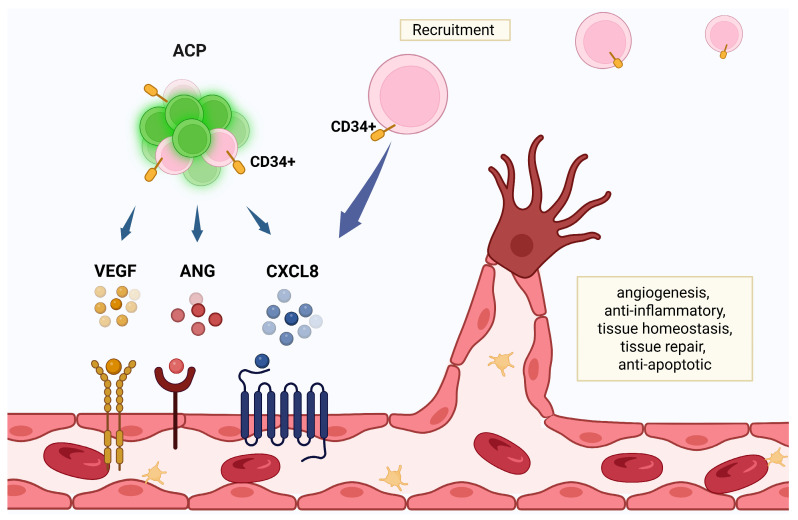
Angiogenic cell precursors (ACPs) potentiate healing through expression of tissue regeneration factors such as the chemokine interleukin-8 (CXCL8), vascular endothelial growth factor (VEGF), and angiogenin. In addition to the robust presence of CD34+ in ACPs, the expressed CXCL8 recruits peripheral CD34+ precursor cells, further supporting angiogenesis. Created in BioRender. Tuchman, K. (2025) https://BioRender.com/w2cthsg.

ACPs exhibit a high degree of CD34+ expression. Whilst there is variability among donors, the published average of one such product of CD34+ cell count in each ACP clinical treatment is 6.0 ± 1.3 × 10^6^. ACPs are programmed to form blood vessels and express tissue regeneration factors such as CXCL8, VEGF, and angiogenin. Additionally, the ACP expression of CXCL8 results in the recruitment of peripheral, perivascular CD34+ cells [20] (Figure 2). VEGF and angiogenin, through the promotion of angiogenesis, increase nutrient supply to damaged brain regions. In the post-stroke condition, enhanced angiogenesis is closely associated with improved neurological and functional outcomes where there has been the simultaneous restoration of the neurovascular unit and the BBB [91,92].

## 8. Activation of NF-κB—The “Learning Molecule”—By CXCL8: NF-κB Is Essential to Synaptogenesis, Neuritogenesis, and Learning

The authors emphasize the importance of the increased activation of NF-κB in the interface between the brain and artificial intelligence and in the context of potential machine learning. Termed by Snow as the “learning molecule” [93], NF-κB is found in highest concentrations in the post-synaptic region, serving itself as a signaling molecule. Once activated, it serves as a transcription factor to activate gene expression and mRNA synthesis of the proteins necessary for dendritic spine formation, synaptogenesis, and neuritogenesis, thus exerting profound influence upon all of the processes of neural plasticity required for long-term memory (LTM) storage. In the development of a machine learning model, the BCI will channel signal acquisition and analysis by AI. The subject’s thought or intention undergo algorithmic apportioning to category and translation into actionable command. The promotion of neural plasticity and memory storage by NF-κB becomes the neurophysiological substrate for an adaptable machine learning model.

NF-κB is ubiquitous in the cytosol of neurons in the cortex, amygdala, and hippocampus. Constitutively present in neurite projections, and essential for synaptic plasticity, NF-κB proteins alone are not sufficient to induce neurite process formation [45], but play a central role in neurite outgrowth, plasticity, and neural cell longevity. The high level of expression of CXCL8 by ACPs [20] results in the activation of cytosolic NF-κB, and its translocation to the nucleus. In the process of memory formation, NF-κB is activated by synaptic activity, and acts as a messenger from synapse to nucleus [93,94].

Kandel laid the underpinnings of the molecular basis of the storage of learned information and memory consolidation, a process involving hippocampus-dependent molecular processes, which include gene transcription [95]. Synaptic plasticity is the primary cellular mechanism for the storage of LTM in the nervous system. Long-lasting neuronal plasticity and LTM require specific gene transcription and the de novo synthesis of m-RNA and protein [93]. Various families of transcription factors have been explored in the context of forming memory: Cyclic AMP response Element Binding Protein (CREB), activating protein 1 transcription factor, CCAAT/enhancing binding protein, and Early Growth Response transcription factor (EGR). However, NF-κB responsive genes play a critical role in learning and memory and are essential to all learning-associated neuronal plasticity, long-term potentiation and long-term depression, the remodulation of dendritic spine and the formation of new synapses (synaptogenesis), and the outgrowth of axons and dendrites (neuritogenesis) [96,97,98,99,100,101,102,103,104,105,106,107,108,109,110]. The NF-κB signaling pathway regulates transcription in the hippocampus during memory reconsolidation.

The transcriptionally active NF-κB dimers are the levers of control for various programs of genetic expression, thus controlling broad gene expression programs which are stimuli-specific [111]. NF-κB resides ubiquitously in the cytosol as an inactive form of three protein subunits—a transcription factor dimer and an inhibitory subunit, IκB. In neurons, the most common combination of subunit is p65 (also referred to as RelA), P50, and IκBα [112,113,114].

**Figure 3:**
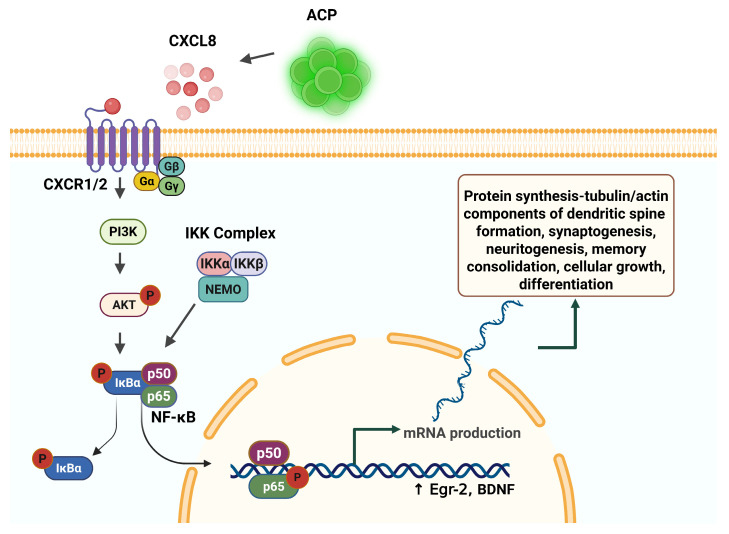
Interleukin-8 (CXCL8) is expressed by angiogenic cell precursors (ACPs), and activates the canonical NF-κB pathway, resulting in gene transcription and protein synthesis necessary for memory formation and consolidation. Created in BioRender. Tuchman, K. (2025) https://BioRender.com/u2icrk5.

In the canonical mechanism of NF-κB activation, the phosphorylation of the inhibitory IκB subunit by IκB kinases (IKK) results in ubiquitination and the proteasomal degradation of the IκB subunit, and the release of the active form of the NF-κB factor dimer to translocate to the nucleus (Figure 3). In the non-canonical control mechanism, IKKα directly modifies chromatin structure by the phosphorylation of histone H3, triggering the acetylation of histone H3 by interacting with the CREB Binding Protein [115,116].

Synaptic activity regulates NF-κB subcellular distribution, DNA binding activity, and transcription [99]. The list of signals activating NF-κB includes the excitatory neurotransmitter glutamate, depolarization, CXCL8, NGF, pro-inflammatory cytokines IL-1 and TNFα, kainate, amyloid β peptide, brain injury, and oxidative stress [41,117,118,119]. NF-κB family members are preformed proteins, the activation and activity of which are regulated to a large extent by post-translational modification, as opposed to induction of their synthesis [120,121].

Synaptic plasticity is regulated by the dynamic balance of excitatory and inhibitory neurotransmissions. Stimulus-driven post-synaptic glutamate results in progressive post-synaptic depolarization. Opposing the excitability is the release of GABA by inhibitory GABAergic neurons, which affect the hyperpolarization of the post-synaptic neuron [122]. The strength of a memory is related to the level of motivation and training, and the epigenetic mechanisms by which its gene expression is subjected. The NF-κB/Rel family helps to modulate the contested terrain of inhibitory interneuron function versus excitatory neuronal function through its effect on GAD65. The loss of neuronal NF-κB results in hyperexcitability and enhanced long-term potentiation [122].

Transcription factor NF-κB, in its role of recruiting histone acetyltransferases and histone deacetylase (HDAC) enzymes at the regulatory regions of target genes, plays a pivotal role in the consolidation and persistence of object recognition memory [123]. NF-κB epigenetically regulates memory consolidation by targeting the transcription factor EGR-1 (also known as Zif268). NF-κB upregulates EGR-1 at the promoter region in the hippocampus, resulting in the increased acetylation of histone H3 [93,115,124]. Histone acetylation by histone acetyltransferases makes DNA accessible to the apparatus of transcription; histone acetylation, methylation, or phosphorylation are involved in long-term plasticity and memory consolidation [123,125]. The inhibition of NF-κB prevents memory consolidation [126].

CaMKII, an abundant synaptic signaling molecule essential for memory formation and metaplasticity, is only induced during the consolidation of a persistent form of memory. CaMKII has four isoforms, of which the delta isoform is regulated by NF-κB after strong training [127]. In particular, the p65 monomer of NF-κB regulates histone acetylation during strong memory consolidation, achieving high levels of H3 acetylation in the NF-κB regulatory region of CaMKIIδ promoter region. The inhibition of NF-κB-dependent histone acetylation impairs persistent memory, and HDAC inhibition renders memory more persistent. Moreover, a weak memory due to weak training, which is unable to generate sufficient gene transcription, can be enhanced by HDAC inhibitors and transformed into a stronger memory [123].

## 9. NF-κB Signaling Pathway Regulates Transcription in Memory Reconsolidation

The period of memory recall is one in which memories are susceptible to alteration and requires a process of reconsolidation after retrieval [108,128]. To enable memory reconsolidation, IKKα activity is necessary to regulate gene transcription and chromatin structure in the hippocampus. The retrieval of contextual conditioned fear memories activates the NF-κB pathway via IKKα, and the subsequent upregulation in hippocampal NF-κB signaling activity increases histone H3 phosphorylation and acetylation. IKK inhibition blocks the regulation of both chromatin structure and NF-κB DNA binding during memory reconsolidation; this inhibition blocks contextual conditioned fear memory reconsolidation and histone H3 post-translational modifications in the hippocampus after memory retrieval. Conversely, elevating histone acetylation rescues this memory deficit in the face of IKK blockade [115,129,130,131].

The importance of NF-κB has been demonstrated in both invertebrate and vertebrate species. The inhibition of NF-κB in the hippocampus disrupts memory [116]. In the crab *Chasmagnathus*, the habituation of the escape response elicited by a fear stimulus is paralleled by an increased activated form of an NF-κB homolog in brain nuclei and in isolated synapses [96,132]. On the other hand, blocking NF-κB activation with an IKK complex inhibitor prevents the formation of escape memory [133], and blocking NF-κB after memory recall results in amnesia [108]. In murine models, intrahippocampal injections of NF-κB decoy disrupt the reconsolidation of memory [126]. NF-κB is needed for radial maze mastery [134]. The intra-amygdala administration of a NF-κB decoy disrupts the fear-potentiated startle response [135]. The inhibition of the NF-κB function by the overexpression of IkB in the mouse forebrain is associated with the decreased mRNA of the catalytic subunit of protein kinase A, decreased CREB phosphorylation, and decreased spatial memory [105].

## 10. Information Storage Is a Function of Synaptogenesis

Altered strength and connectivity of synapses and dendritic spine morphology underlies neural plasticity [136,137,138,139]. The transcription factor NF-κB is centrally involved in the upregulation of dendritic spines and synaptic density. NF-κB is necessary in the regulation of signals which induce neurite outgrowth during synaptic plasticity and memory. NF-κB influences the morphology and complexity of the dendritic arbors through the regulation of neural cell adhesion molecule, amyloid precursor protein, Tenascin-C, and β1 integrin [93]. Moreover, the p65 subunit of NF-κB is enriched in dendritic spines, where it regulates the density, size, and function of dendritic spines and excitatory synapses, and provides feedback to control spine density and spine morphology. During the learning process, plasticity is manifested by a substantial increase in NF-κB over basal level in a number of dendritic spines and excitatory synapses [140]. The p65-mediated regulation of spine density absolutely depends on the ability of p65 to bind DNA, and to activate the transcription of target genes. Structural plasticity also involves changes in dendrite head size; p65 increases dendrite head size. Larger spine heads and larger synapses correlate with increased numbers of AMPA receptors and increased AMPA receptor-mediated currents. Conversely, a loss of NF-κB function leads to a decrease in dendritic spine density, and diminished spine head size [141,142].

NF-κB upregulates BDNF, which is profoundly manifest in terms of memory consolidation, cellular growth, differentiation, and neurogenesis [143]. Thus, the high levels of CXCL8 expressed by ACP activate NF-κB, and this effect is of profound importance to the potential for learning and memory storage.

## 11. NSCs Favor the Non-Inflammatory M2 Phenotype, Resulting in Less Inflammation and Less Scarring

**Figure 4:**
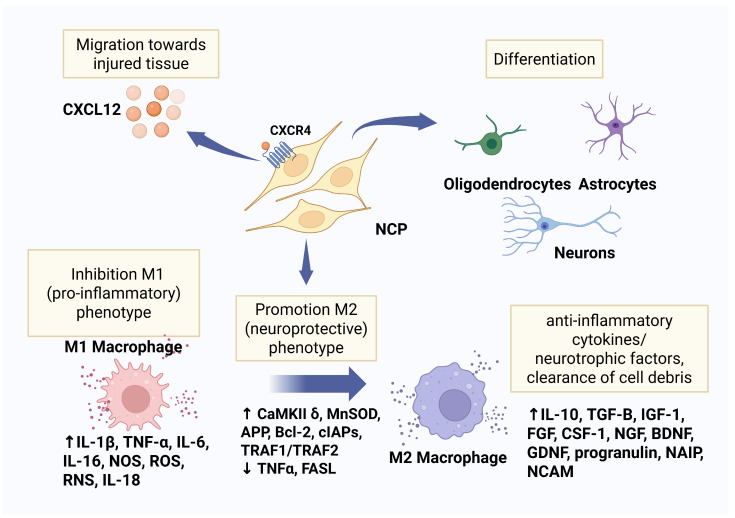
Neural progenitor cells (NCPs) have CXCR4 receptors and migrate towards CXCL12 released by injured tissue. NCPs promote the M2 (neuroprotective) macrophage phenotype, which release anti-inflammatory factors. The NCPs differentiate into neural, glial, or oligodendrocytic cells. Created in BioRender. Tuchman, K. (2025) https://BioRender.com/36j1vy7.

The grafting of human NSCs intracerebrally attenuates microglial/macrophage activation. In preclinical stroke studies, the intraparenchymal administration of NCPs is associated with a decrease in the number of activated microglia [144]. NCPs suppress the activation and proliferation of pro-inflammatory T cells [145]. Moreover, NSCs signal the preferential adoption of the M2 phenotype, thus promoting neuroprotection and tissue healing, and decreasing deleterious inflammatory changes and scarring in areas of CNS injury (Figure 4) [146,147]. The M2 phenotype promotes axonal regrowth in the setting of traumatic injury [148]. Ubiquitously distributed throughout the CNS, microglia, depending on their activation status, can be pro-inflammatory and cytotoxic—the M1 “classical” phenotype or conversely the neuroprotective M2 “alternative” phenotype. Oxidative stress, low-grade inflammation, injury, and neurotoxin exposure result in microglial priming toward the inflammatory and harmful macrophage M1 phenotype. Pathogenic particles from damaged cells trigger the release of pro-inflammatory cytokines IL-1β, TNF-α, IL-6, IL-16, IL-18, nitrous oxide, ROS, and reactive nitrogen species, reciprocally amplifying microglial activation, and resulting in neuron vulnerability and neuronal death [149,150,151]. This inflammatory response may be amplified through the recruitment of additional cells by pro-inflammatory cytokines [152,153]. The M1 and M2 phenotypes represent a continuum, and microglia may move from one state to the other [154]. Though neuro-inflammation serves as a defense mechanism to protect the brain by removing or inhibiting diverse pathogens, sustained inflammation inhibits regeneration [155]. NCPs lessen scarring by promoting the adoption of the M2 phenotype of microglia and macrophages. M2 microglia aid the phagocytosis of cell debris and misfolded proteins, promote extracellular matrix reconstruction and tissue repair, and support neuron survival by the release of neurotrophic factors [152]. The M2 phenotypical macrophages release anti-inflammatory cytokines—IL-4, IL-10, IL-13, transforming growth factor-β, insulin-like growth factor-1, fibroblast growth factor, colony stimulating factor 1, and neurotrophic growth factors—BDNF, neurotrophins, and GDNF, inducing a condition of neuroprotectivity [156]. While microglia originate from infiltrated yolk sac progenitor cells during early embryonic development, and are maintained independently by self-proliferation, they are also maintained in number by circulating monocytes under disease conditions [157]. Within 24 h of stroke, monocytes from the peripheral circulation adhere to the damaged endothelium of the BBB and infiltrate the CNS to replenish the macrophage count [158].

## 12. Efficacy of NCPs and ACPs

A large body of evidence supports the efficacy of NSCs in the treatment of stroke and other neurological conditions [65,66,67,68,75,159,160,161,162,163,164]. The efficacy of ACPs has been demonstrated in the treatment of cerebral infarction, heart failure, and critical limb ischemia [18,19,65,161,165,166,167]. In preclinical studies, ACP and NCP grafts have demonstrated the ability to restore vascular integrity and improve BBB function [168,169] and to reduce stroke-induced elevated matrix metalloproteinase (MMP)-9 levels [170].

## 13. Safety and Strategic Implications of Intrathecal Delivery of Autologous ACPs/NCPs to Target Tissue

The authors propose the staggered intrathecal administration of ACPs and NCPs via lumbar puncture, with the goal that these cells will, by normal CSF flow mechanisms, reach the target sites of electrode placement in adequate numbers to minimize scar formation around the electrodes, improve the survival of ambient host neural tissue, and create the optimal molecular milieu to enhance functional signaling and ultimately machine learning.

The safety and efficacy of intrathecal injections of ACPs and NCPs have not been tested in the BCI paradigm. However, in clinical practice, there have been no complications related to the cell product in the ACP treatment of heart failure or critical limb ischemia. One mortality in the heart failure study of Schubart et al. resulted from a technical error in the transcatheter cell transplantation in a patient with silent MI [18,19,166,167]. There has been a plethora of studies that demonstrate the safety of the intrathecal administration of the stem cells [171,172,173] and of intracerebral injections of stem cells [159,174]. However, the optimization of stem cell application in clinical practice is in its infancy. Broadly, there are three paramount issues in providing stem cell therapy.

### 13.1. Appropriateness of Cell

Firstly, the determination of the most appropriate cell for the disease process. While preclinical work has been performed applying induced pluripotential stem cell therapy in stroke, spinal cord injury, Alzheimer’s disease, Parkinson’s disease, and ALS, these cells can be complicated by neoplasia or other undefined genetic polymorphisms or epigenetic changes that will be carried into the final cell product [175]. Embryonic stem cells are highly pluripotent and also have a higher tumorigenic risk [176]. However, ACPs and NCPs, as derived at a later state of differentiation, have no known risk of neoplasia. Autologous ACPs and NCPs also have the benefit of zero immunogenicity, with no risk of rejection, and no need for immunosuppressive agents [171]. Whilst the facility of harvesting larger numbers of mesenchymal stem cells is attractive, the biomolecular profiles of ACPs and NCPs appear to be appropriate for the treatment of electrode-related scarring and cell loss, and, theoretically, in the provision of an optimal milieu for learning.

### 13.2. Targeted Delivery of ACPs/NCPs

The second primary issue relates to the means of the most targeted delivery of a therapeutic quantity of cells to the area of injury. Stem cell delivery may utilize intravenous or intra-arterial injections, intrathecal or intra-cerebro-ventricular injections, or direct intracerebral injections into brain parenchyma. Intracerebral transplantation is effective in depositing a large number of cells to a specific location. However, the cells remain more localized and migrate only short distances. The process of intracerebral injection is technical, expensive, and associated with complications of headaches, hemorrhages, seizures, and injuries to the surrounding tissue [177].

The intravenous injection of bone marrow-derived mesenchymal stem cells has demonstrated poor cell homing to the brain. This is in part due to the blood–brain barrier, which is impermeable to the majority of stem cells delivered by endovascular injections. In the absence of inflammation, stem cells generally do not cross the intact blood–brain barrier and are more likely to be mechanically trapped in peripheral organs [177].

The intrathecal delivery of cells via the lumbar CSF cistern is a common, relatively low-risk procedure, that can be broadly applied and repeated safely [171]. The intrathecal administration of cells has been safely performed in ALS, multiple sclerosis, spinal cord injury, and multiple system atrophy [178,179].

The lumbar puncture is accomplished with local anesthesia and sometimes a sedative. Complications of lumbar puncture include spinal headache for several days (32%) (which is in most cases due to a small CSF leak and is, in the great majority, temporary), nerve injury (rare), infection (rare), and hematoma (0.17%) [180,181]. However, the use of a 25 gauge pencil point needle used in spinal anesthesia allows an ample volume (2 mL per 5 min) to be infused with an almost zero risk of headache. The risk of an allergic reaction to the administered cells is very low given that the cells are autologous and the diluent has not been associated with any reaction.

Intrathecal delivery, by lumbar puncture, directly into the cerebrospinal fluid accomplishes the introduction of cells into a targeted circulation volume of approximately 120–150 mL, with no risk of filtration of cells by the liver, spleen, or lungs. Conversely, after intravascular injection, murine studies have found the majority of mesenchymal stem cells (diameter 15–19 microns) to be trapped in the pulmonary microvasculature within seconds [182].

Each treatment of one available product includes an average of 12.0 ± 4.7 × 10^6^ ACPs (including a minimum of at least 5 × 10^6^ CD31+ bright/AcLDL cells (ACP) and at least 1 × 10^6^ CD34+ cells). The ACP cell number varies with the health of the patient, approximately from 6 × 10^6^ to 30 × 10^6^. Other transplantation studies have demonstrated safety with intracerebral injections of 1.2 × 10^7^, 2.4 × 10^7^, or 7.2 × 10^7^ cells [174].

Intrathecal delivery utilizes CSF dynamics to move the cells to their target(s), which may be many in the context of multiple electrode placement. From the lumbar cistern, the normal flow of CSF circulates up through the base of the skull and over the convexity of the brain, toward the arachnoid villi along the midline superior sagittal sinus, which absorbs the CSF [183]. Intrathecally delivered cells do not have to cross the blood–brain barrier and are able to penetrate the parenchyma in the perivascular space alongside the perforating blood vessels. In the perivascular space, there is mixing of CSF with interstitial fluid, including expressed factors draining from the brain. Normal CSF flow will thus carry injected cells into the proximity of perforating electrodes.

## 14. The NCP Would Be Administered One Week After the ACP. This Is Due to the Increased Time for the Production Time of NCP

The risk of “off-target” effects of intrathecal injection is minimized by the containment of cells within the limited volume of cerebrospinal fluid and central nervous system itself. It is unknown the extent to which the expressed neurotrophic factors, released chemokines, and proteins pass into the blood circulation and exert a corporeal effect.

### Cell Longevity and Function

The third strategic issue relates to the functionality and longevity of survival of the cells once they reach their intended site. The autologous ACPs and NCPs are not subject to any form of immune response or major histocompatibility complex interactions. Preclinical studies in heart failure demonstrated the robust survival of ACPs for at least 1 month after transplant [17].

Grafts of human NSCs (total 1.60 × 10^5^) transplanted to the spinal cord of rodents with lesions showed differentiation into GABAergic neurons which formed synapses in the ventral horn at 6 weeks [184]. In clinical trials in the U.S. for the treatment of paralysis due to stroke, 12–24 months prior to transplantation of NSCs (fetal-derived, NSI-566), all subjects demonstrated evidence indicating cavity filling by new neural tissue at 2 years [159,174]. The survival of NCPs after intrathecal injection is unknown.

Bone marrow-derived mesenchymal stem cells are abundant, possess low immunogenicity, produce numerous paracrine cytokines, promote capillary growth, and are associated with few complications. However, implantation may be impaired in conditions of a hypoxic microenvironment [185]. The injection of mesenchymal stem cells into the blood stream may result in a short existence, with fewer than 1% of cells surviving 4 days [186,187]. Notwithstanding the fact that the CSF is immune-privileged, class II MHC molecules and the microglia can interact with allogeneic cells. Despite the absence of the major histocompatibility complex class II antigens, mesenchymal stem cells may still suffer immune rejection from alloreactive antibodies [188]. Fresh autologous cells have superior efficacy to stored allogeneic mesenchymal stem cells [189].

## 15. Limitations

We emphasize there is as yet no experimental data on ACPs and NCPs in the context of the BCI paradigm. The proposed appropriate dosing strategy has been based around the number of cells in a blood draw of 250 mL but appears commensurate with cell numbers in other studies. Patients would probably need annual treatments of ACPs and NCPs to maintain the interface. Presently, the cost of producing the cells is high, though it is anticipated that with automation, cell production costs should be substantially reduced. ACPs and NCPs are not currently FDA approved for any application, including the BCI, but the states of Florida and Utah have approved the use of ACPs for the treatment of cardiomyopathy and chronic limb ischemia. Other states are likely following.

## 16. Conclusions

The application of artificial intelligence through the brain–computer interface is proving to be one of the great advances in neuroscience today. Notwithstanding the quality of the electrode–brain interface, the immune response and triggering of programmed cell death—apoptosis—are limiting factors to long-term success. The biomolecular strategy provided by the intrathecal delivery of ACPs and NCPs is proposed as a solution to foreign body reaction, scarring, apoptosis, and decreased signaling. ACPs and NCPs express CXCR4 receptors, which have high specificity for chemokine ligand CXCL12, the axis of which promotes the migration of the cells to areas of injury. ACP-associated CD34+ cells and the released factors of VEGF and angiogenin promote angiogenesis. Immunomodulation due to IL-8 attraction of NK cells, and the NCP adoption of the non-inflammatory M2 phenotype, limit the inflammatory changes that accompany CNS injury. Most importantly, NCP’s secretion of NGF, and the elevated levels of IL-8(CXCL8) activated transcription factor NF-κB, serve to generate anti-apoptotic proteins and to affect a central role in neurite outgrowth and neural plasticity, learning, and memory storage. The intrathecal administration of ACPs and NCPs by lumbar puncture, repeated as necessary, is hypothesized to be a safe, effective means of establishing a living substrate and maintaining the signaling characteristics of the inserted electrodes at the BCI.

## Data Availability

No new data were created or analyzed in this study.
